# Empirical Modeling of Zn/ZnO Nanoparticles Decorated/Conjugated with Fotolon (Chlorine e6) Based Photodynamic Therapy towards Liver Cancer Treatment

**DOI:** 10.3390/mi10010060

**Published:** 2019-01-17

**Authors:** Seemab Iqbal, Muhammad Fakhar-e-Alam, M. Atif, Nasar Ahmed, Aqrab -ul-Ahmad, N. Amin, Raed ahmed Alghamdi, Atif Hanif, W. Aslam Farooq

**Affiliations:** 1 Department of Physics, Government College University, Faisalabad 38000, Pakistan; seemabiqbal11@hotmail.com (S.I.); gourmani5@yahoo.com (N.A.); 2 Key Laboratory of Magnetic Materials and Devices & Division of Functional Materials and Nanodevices, Ningbo Institute of Materials Technology and Engineering, Chinese Academy of Sciences, Ningbo 315201, China; 3 Department of Physics and Astronomy, College of Science, King Saud University, Riyadh 11543, Saudi Arabia; muhatif@ksu.edu.sa (R.a.A.); wafarooq@hotmail.com (W.A.F.); 4 Department of Physics, University of Azad Jammu and Kashmir, Muzaffarabad 13100, Pakistan; fakharphy@outlook.com; 5 School of Physics, Dalian University of Technology, Dalian 116024, China; a.aqrab4469@gmail.com; 6 School of Microelectronics, Dalian University of Technology, Dalian 116024, China; 7 Botany and Microbiology Department, College of Science, King Saud University, Riyadh 11543, Saudi Arabia; ahchaudhry@ksu.edu.sa

**Keywords:** ZnO nanoparticles, photodynamic therapy, photosensitizer, hepatocellular model, bio toxicity

## Abstract

The current study is based on Zn/ZnO nanoparticles photodynamic therapy (PDT) mediated effects on healthy liver cells and cancerous cells. The synthesis of Zn/ZnO nanoparticles was accomplished using chemical and hydrothermal methods. The characterization of the synthesized nanoparticles was carried out using manifold techniques (e.g., transmission electron microscopy (TEM), X-ray diffraction (XRD), and energy dispersive X-ray spectroscopy (EDS)). In order to study the biotoxicity of the grown nanoparticles, they were applied individually and in conjunction with the third generation photosensitiser Fotolon (Chlorine e6) in the in vivo model of the normal liver of the Wister rat, and in the in vitro cancerous liver (HepG2) model both in the dark and under a variety of laser exposures (630 nm, Ultraviolet (UV) light). The localization of ZnO nanoparticles was observed by applying fluorescence spectroscopy on a 1 cm^2^ selected area of normal liver, whereas the in vitro cytotoxicity and reactive oxygen species (ROS) detection were carried out by calculating the loss in the cell viability of the hepatocellular model by applying a neutral red assay (NRA). Furthermore, a statistical analysis is carried out and it is ensured that the *p* value is less than 0.05. Thus, the current study has highlighted the potential for applying Zn/ZnO nanoparticles in photodynamic therapy that would lead to wider medical applications to improve the efficiency of cancer treatment and its biological aspect study.

## 1. Introduction

Cancer is one of the leading causes of death in the present advanced era. There is a basic need to develop more reliable and comprehensive methodologies for its treatment and diagnosis. Photodynamic therapy is a special type of technique that is used to investigate the treatment of solid malignant tumors. The basic principal of this therapy is exposure of red light with a specific wavelength (near 630 nm) with a suitable photo sensitizer on malignant tissues. The absorption of red light is an important property for starting the photodynamic action within the tumor site. In this technique, a high, intense dose of radiation destroys the maligned tumor. This therapy has significant advantages over other traditional therapies (chemotherapy) because of the localization of particular targeted areas. Hence, the chances of damaging the healthy cells in a body are significantly less. This therapy is more suitable for those cancers spreading throughout the body. In this technique, doses of radiation deliver only to a particular tumor site, hence these radiations do not travel throughout the body to destroy the normal cells.

Currently, metal oxides, especially zinc oxides, have received more attention because of their unique physiochemical properties. ZnO is a piezoelectric, direct, and wide band gap (3.39 eV) semiconductor material. It has wide range of applications especially in opto-electronics, biomedical physics, and nanopiezotronics. Moreover, it has very interesting biological assets, such as anti-cancer and anti-bacterial properties, which can be obtained by controlling the morphology of 3D self-assembled micro/nanostructures of ZnO [1]. Increasing the use of ZnO nanoparticles (NPs) necessitates an improved understanding of their potential impact on the environment and on human health.

The liver cancerous HepG2 cell line is used for in vitro and in vivo studies [2,3,4]. The HepG2 cell line is morphologically similar to the parenchyma cells, and has the ability to synthesize the plasma proteins and growth factors (e.g., protoporphyrine (PpIX)) [5,6]. Moreover, the tumorous liver undergoes the enhanced action of multidrug resistance (MDR) genes (e.g., P-Gp), which provides hindrance to drug accumulation in tumorous cells. Thus, to overcome this problem, nanoparticles of ZnO are used. The topical application of aminolevulinic acid (ALA) causes an accumulation of PpIX in the HepG2 cell line. The PpIX conjugates with ZnO NPs and surpasses the membranous efflux proteins and undergoes accumulation inside the tumour, and accomplishes the requirement of photodynamic therapy (PDT), commonly known as photo dynamic therapy (PDT) [7]. PDT is a less invasive and more reliable technique for the eradication of tumours using a photosensitiser conjugated with nanoparticles and light of a suitable wavelength [8].

**Figure 1:**
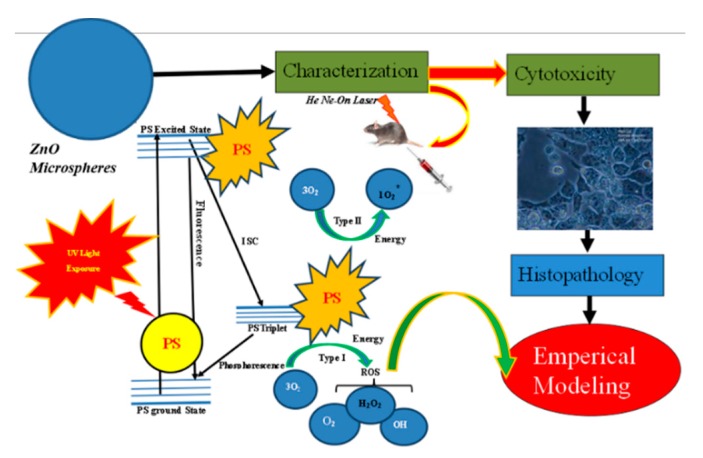
Schematic illustration of ZnO nanoparticles-based photodynamic therapy (PDT) towards an in vivo and in vitro model.

In the current work, the prime focus of the work is to trace the biological activity of ZnO nanoparticles towards an in vitro and in vivo model. The advantages of these synthesis methods compared to other conventional methods are the simplicity of the setup and the limited tendency of particles to aggregate, along with a high-quality homogeneous crystal structure. Moreover, the hydrothermal method is an environmentally friendly approach, because it does not require compatible solvents or some authentic procedures. Figure 1 depicts the overall summary of the work of the current conducted experiment.

## 2. Materials and Methods

### 2.1. Synthesis of ZnO Nanoparticles

The synthesis was performed via two approaches, chemical and hydrothermal [9]. In the chemical approach, a solution of zinc sulfate ZnSO_4_·7H_2_O in deionized water (DI) was prepared by using a magnetic stirrer until all of the zinc sulfate was completely dissolved. The molarities of the zinc sulfate ZnSO_4_·7H_2_O and ammonium hydroxide NH_4_(OH) (99.9%, Fisher Scientific, Hampton, NH, USA) were set and the molar ratio of the Zn^2+^/NH_3_ solution ranged to 1:10. A cleaned laser indented piece (1 cm × 1 cm) of boron doped p-type silicon (111) (MTI Corporation, Richmond, CA, USA) was placed in the flask containing solution of Zn^2+^/NH_3_, and 1.0 g of pure zinc powder was added into the solution. The whole system was transferred to the oven for uniform heating around 95 °C for 15 min, and then the sample was allowed to cool down to room temperature. The prepared sample was washed by dipping it in DI water, and was dried in air at 150 °C for 5 min.

In the hydrothermal approach, initially, zinc sulfate ZnSO_4_·7H_2_O was dissociated in water to produce zinc (Zn^2+^) and sulfate ions (SO_4_^2−^). The addition of ammonia (NH_3_) caused a change in the pH of the solution and the Zn(OH)_2_ started to precipitate. The addition of NH_3_ to this solution produced white gelatinous Zn(OH)_2_ precipitates because of the reaction of Zinc (Zn^2+^) ions with the aqueous ammonia. After adding ammonium hydroxide in an excess amount, the zinc complexes started to develop. The precipitates started to dissolve, and the solution became clear when the molar ratio of Zn^2+^/NH_3_ reached 1:4 or higher. Upon heating at 95 °C, the Zinc complexes dissociated and dehydrated to form ZnO. The addition of zinc produced Zn/ZnO composite structures and formed Zn/ZnO nanoparticles in the solution.

### 2.2. Cell Culturing and Labeling

In the cell culturing process, the HepG2 cell line was cultured in tissue-culture plastic flasks (Nunc, Wiesbaden, Germany) in Minimum Essential Medium (MEM) with Hanks salts, also supplemented with 10% fetal bovine serum (FBS), 2 mL glutamine, and with some nonessential amino acids. Moreover, for a suitable connection with the substratum, the cells were incubated for 24 h at 37 °C. The cells were also sub-cultured two or three times in a week. After that, the cells were harvested via trypsin 0.25% once they reached the confluence of 75–85%. The HepG2 cells with a concentration of 1 × 10^5^ cells/well were incubated with different concentrations ranging from 10–250 µg/mL of ZnO nanoparticles in dispersed solutions [10]. In a parallel experiment, the cells were treated with the same concentration of ZnO nanoparticles with a Fotolon (Chlorine e6) complex, and were exposed with a 20 J/cm^2^ dose of UV lamp light using a UV lamp. Each concentration was labeled/exposed in five wells, and the data were repeated three times in a routinely cultured cell model. After the assessment of a suitable time of incubation, the next step was laser exposure (630 nm of diode laser, having 80 J/cm^2^ most suitable values were optimized for in vitro cell study) [11,12].

### 2.3. Animals

Male Wistar rats weighing between 150–300 g were used in this experiment. The animals and cells were maintained according to the guidelines of the committee on care, and the use of cell laboratory and research center for ethics (West China Hospital, Sichuan University, Huaxi Campus, China).

### 2.4. Photosensitizer

We used Fotolon as a photosensitizing drug for the animal and cell uptake for employing different steps of PDT. The optimized concentration of pure Fotolon and Fotolon with ZnO nanoparticles rangeing from 0–250 µg/mL were exposed to HepG2 cells for the assessment of cell viability loss in the dark and under various forms of light (visible/UV light). Moreover, the scientific phenomena of the cell killing process because of Fotolon individually as well as in the form of a complex were depicted in experimental scheme. 

### 2.5. Photodynamic Therapy (PDT) Procedure

In the current experimental study, the actual biodistribution, biodegradation, and toxicity of the ZnO nanoparticles for in vivo and in vitro cancerous liver models were investigated. In this experimental scheme (Figure S1), 25 animals (Wistar rats weighing between 150–300 g) were selected and divided into five groups. The ethical recommendation/approval was granted (Wistar rats and HepG2 cells were maintained according to the guidelines of the Committee on Care and Use of Cell Laboratory and Research Center for Ethics (West China Hospital, Sichuan University, Huaxi Campus, China)). After anesthesia, the rats were operated on and 10–250 µg/mL working solutions of ZnO individual nanoparticles and ZnO nanoparticles along with Fotolon with respect to the body weight was injected through the vena cava and directed into the liver site. Five groups of animals were studied in a parallel study, that is, in the first group, a liver model was taken as a reference; in the second group, the ZnO nanoparticles toxicity was elucidated; in the third group ZnO nanoparticles phototoxicity was used (red laser light exposure, λ ≈ 630 nm), in the fourth group, a ZnO and Fotolon PDT procedure was employed in the presence of UV lamp light (λ ≈ 240 nm) with an energy of 20 J/cm^2^ for 2–3 min; and similarly, in the fifth group, a ZnO and Fotolon PDT procedure was employed in the presence of a red laser light (λ ≈ 630 nm). After 24 h, the animals were scarified and the PDT treated livers were removed and their histopathological analysis was performed. 

### 2.6. Fluorescence Spectroscopy Analysis

**Figure 2:**
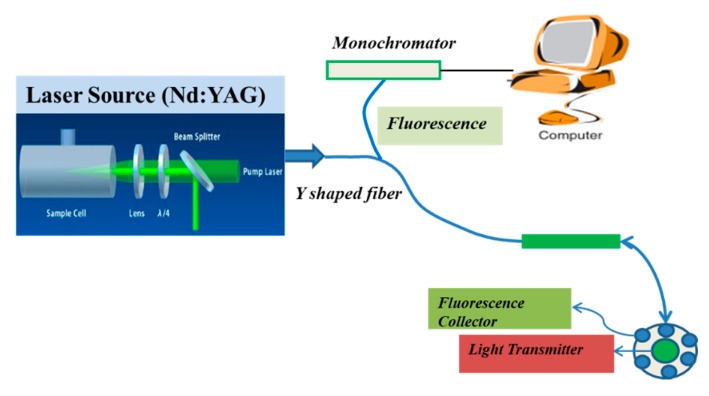
Schematic diagram of fluorescence spectroscopy.

In this piece of the experimental step, the uptake of the ZnO nanoparticles was confirmed by applying fluorescence spectroscopy. The fluorescence spectroscopy was performed for the actual depth data of the tissue via fluorescence spectroscopy analysis. The strategy is a newly developed technique based on placing green emission Nd:YAG as an exciting source for the reliable spectroscopic signature of hidden parts of a live tissue model. Basically, the desired light was transmitted to collect the internal fluoresce of the liver tissue for chemical uptake presence in the targeted territory. Simultaneously, six optical detectors were fixed around the center (transmitter one) and were interconnected for fluorescence collection by performing y-shape optical fiber using Nd:YAG (with 532 nm of second harmonic green laser of glow as excitation source) to collect fluorescence between the visible and near infrared light (540–850 nm). The whole schematic experimental set up is shown in Figure 2.

### 2.7. Cellular Viability

The cell viability of the ZnO nanoparticles’ exposed cells were assessed by applying the standard protocol of a neutral red assay (NRA) analysis [2,3,4,5]. In the first step, the cells were seeded in 96-well plates and were exposed to a different concentration of ZnO nanoparticles dispersion, with a mixture of Fotolon in the absence and presence of laser light exposure (630 nm and UV light). After 24 h of cell incubation with ZnO nanoparticles at optimal concentration, 50 μL of neutral red assay (50 mg/mL) was incorporated in the treated cultured plate and incubated for 3 h [2,3,4,8,9]. The medium was removed and the cells were washed with 40% formaldehyde and 10% cacl_2_ (*v*/*v*, 4:1). In the next step, a complex of 45% ethanol and 15% acetic acid (1:1) was assimilated to extract NR. In a further step, the NR-mixed plate was shaken for 50 s and kept free for 15 min. The absorbance of the incorporated dye was gently examined at 510 nm. The quantification of solubilized dye was statistically analyzed with the living cell numbers, as formulated below [7,8,9]:

(1) $$Percent\;\left(\%\right)\;cell\;viability=\frac{Mean\;Absorbance\;of\;HepG2\;treated\;cells}{Mean\;Absorbance\;of\;Controlled\;cells}\times 100$$

### 2.8. Characterization Techniques

The topography of the prepared sample (synthesis of sample preparation was elaborated in Section 2.1) was studied using (JSM-6510LV, Jeol, Tokyo, Japan) transmission electron microscopy (TEM) operated at voltage 25 kV. The energy dispersive X-ray spectroscopy (EDS) system was used to study the composition of the different elements present in the prepared sample. The structural properties of the newly synthesized Zn/ZnO nanoparticles were further characterized using an X-Ray diffractrometer (Pan Analytical, XPERT-PRO system, Malvern, UK), operated at a voltage of 40 kV and a current of 40 mA, using a CuK_α_ (λ = 1.54 Å) radiation source. The diffraction patterns were recorded at a small grazing incident angle of 4°.

### 2.9. Mathematical Modelling Analysis

The method of least square error using Matlab version 2016 is applied to the experimental data in order to have a mathematical model of the dependency and verification of the experiments. The value of correctness of fit is evaluated by taking the value of R-square = 0.9862, which indicates the correctness of the math model. After which, the surface plot of the experimental values is plotted to calibrate the cell viability with the increase of concentration effect of ZnO and Fotolon into the cells. 

## 3. Results and Discussion

### 3.1. X-Ray Diffraction (XRD) and Energy Dispersive X-Ray Spectroscopy (EDS) Analysis 

**Figure 3:**
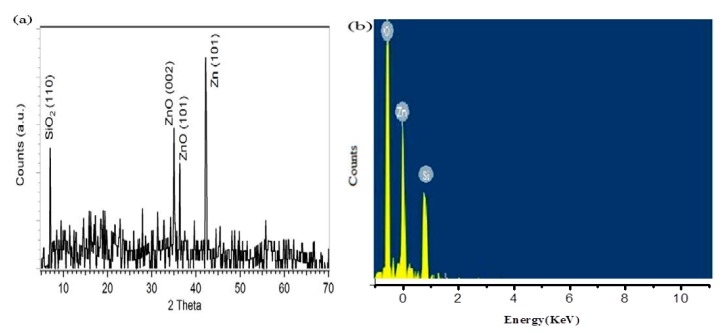
Structural and compositional analysis of Zn/ZnO nanoparticles grown on indented sites. (**a**) XRD peaks. (**b**) Energy dispersive X-ray spectroscopy (EDS) spectrum by hydrothermal route.

The X-ray diffraction analysis gave the information about the crystallography and size of the nanoparticles. The obtained XRD pattern for the prepared ZnO nanoparticles system is displayed in Figure 3a. The prominent peaks of ZnO were noted at 2θ = 34.25° and 2θ = 36.34°, and correspond to the (002) and (101) planes of the hexagonal structure of ZnO, respectively; whereas the strong peak at 2θ = 43.18° belongs to the (101) plane of pure Zn. The identified peak positions and relative intensities of the Zn/ZnO nanoparticles were compared with the (JCPDS) card for ZnO (JCPDS 036-1451) and for Zn (JCPDS PDF #00-0040831). The observed diffraction peaks confirm the high crystallinity of the structure. The diffraction peaks of SiO_2_ and Zn were also observed in the XRD pattern. The Zn peak further confirmed the formation of the Zn/ZnO composite, and the SiO_2_ peak is present because of the Si substrate. The EDX analysis performed for the synthesized nanoparticles is shown Figure 3b, which depicts the peaks belonging to Zn, O, and Si.

### 3.2. Transmission Electron Microscopy (TEM) Analysis

**Figure 4:**
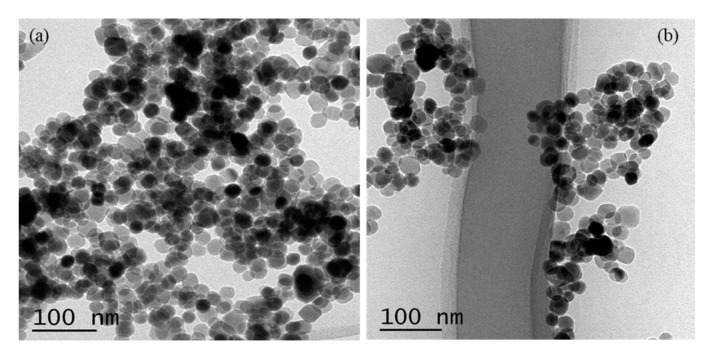
(**a**) TEM morphology of Zn/ZnO nanoparticles. (**b**) Various selective TEM images of Zn/ZnO single nanoparticles.

The TEM images interpret the actual morphology/shape of the nanostructure that was grown. The morphology of the synthesized Zn/ZnO nanoparticles is confirmed by transmission electron microscopy (TEM), as depicted in Figure 4a,b. The TEM images confirmed the growth of Zn/ZnO nanoparticles with varying diameters (10–20 nm), and all of the generated Zn/ZnO nanoparticles are nearly spherical in shape. Figure 4a shows the formation of Zn/ZnO nanoparticles with a mixture of some black and white circles with various morphologies. It depicts the spherical morphology of enlarged nanoparticles, with an approximate diameter of 10 nm diameter.

### 3.3. Absorption Spectrum Analysis 

**Figure 5:**
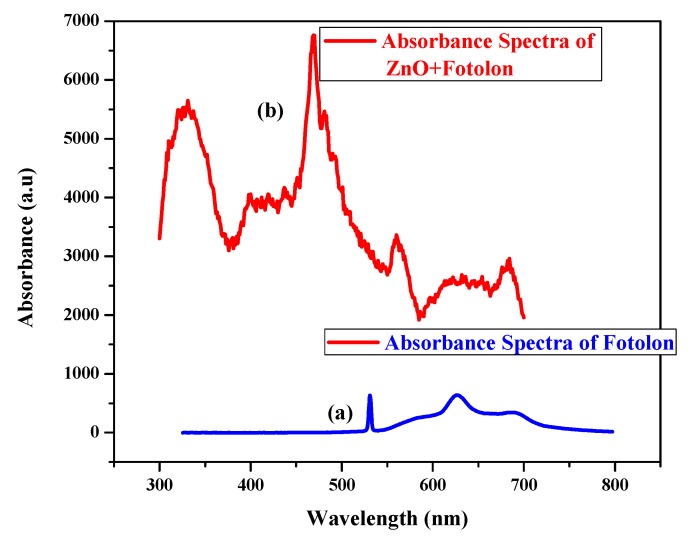
(**a**) Absorption spectra of Fotolon. (**b**) Absorption spectra of ZnO complex with Fotolon.

Tissue absorption spectroscopy plays a key role for effective PDT. Figure 5a,b shows the absorption spectra of a Fotolon and ZnO complex with Fotolon. The peaks are well defined in the respective spectra. Obviously, Figure 5a depicts the chemical signature of the third generation photosensitizer Fotolon^®^ (from 0 to 24 h of incubation), as the first back scattered peak and second dominant peak are well defined in the visible region at about 625 nm of the red wavelength. Similarly, Figure 5b represents the sandwich signature of the ZnO complex with Fotolon. It is obvious from Figure 5b that the UV absorption peak, which is prominent, corresponds to the ZnO nanoparticles, and the rest of the peaks relevant to Fotolon. 

### 3.4. Percent Cell Viability and Photo Toxicity of Zn/ZnO Nanoparticle After PDT

**Figure 6:**
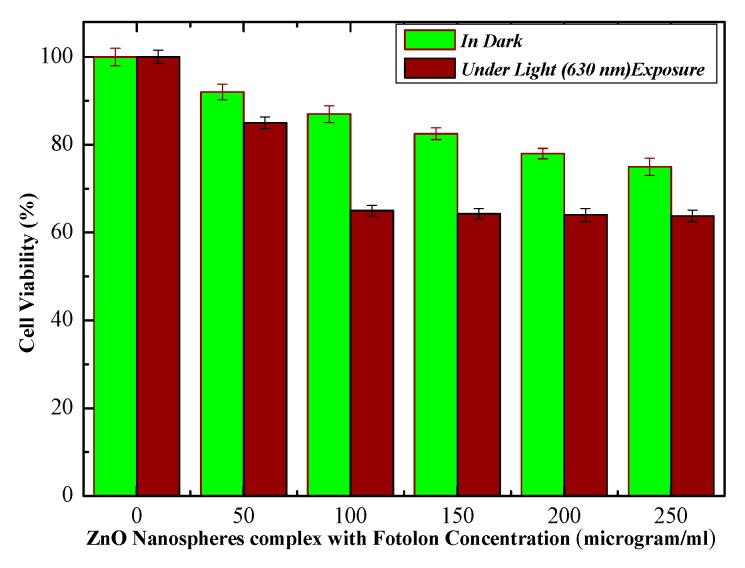
Percent cell viability in dark and presence of light (λ ≈ 630 nm).

**Figure 7:**
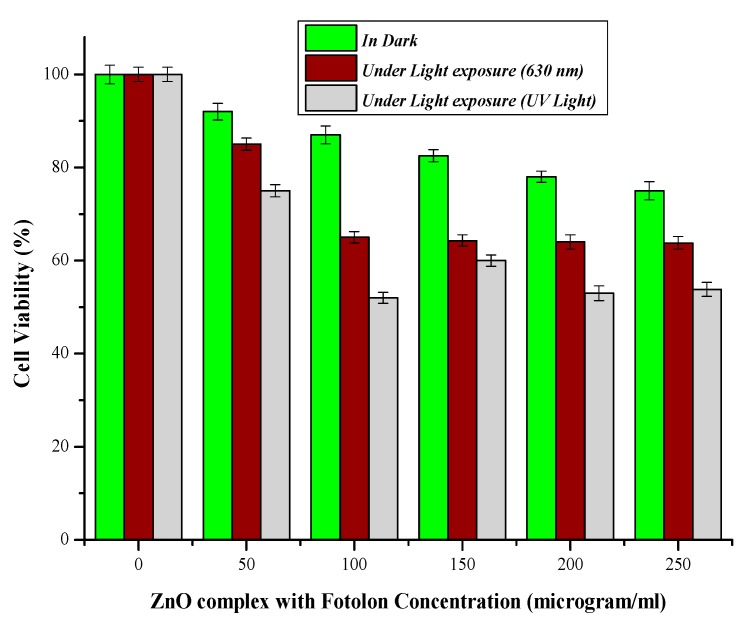
Percent cell viability in dark and presence of light (λ ≈ 630 nm) and (UV-light).

The cell viability losses under laser light (λ ≈ 630 nm) exposure and in dark conditions are displayed in Figure 6. The live cells trend decreases by steadily increasing the ZnO nanoparticles complex with the Fotolon (ce6) concentration and light presence. After an optimal time of span, 37% cell viability losses were found in the case of ZnO and Fotolon, and 630 nm of light exposure; but this loss reaches about 25% in a dark condition, and about 47% cell losses were assessed while exposed with UV light. A significant difference between the exposure under darkness and under 630 nm of light is noticed with a 250 µg/mL of ZnO and Fotolon concentration. Figure 7 demonstrates the dependence of the percentage loss of the cell viability (%) after treatment with ZnO nanoparticles and Fotolon against UV light doses of 20 J/cm^2^, which shows a dependency of the cell viability on the concentration of Zinc nanoparticles complex with Fotolon under UV illumination. The differentiation can clearly be seen between the cell viability due to exposure to ZnO and Fotolon nanoparticles in the dark and under a suitable dose of 20 J/cm^2^ of UV light after labeling with a HepG2 cellular model. It is worth mentioning that we have previously reported on the different cell lines that were labeled with ZnO nanomaterials with different sizes and morphologies, for example, zinc oxide nanorods (ZnO nanorods (NRs)), zinc oxide nanoparticles ZnO NPs, zinc oxide nanoporous (ZnO NP_S_), zinc oxide nano flakes (ZnO nanoflakes (NFs)), zinc oxide Nanotubes (ZnO nanotubes (NTs)), and zinc oxide nano wells (ZnO nanowires (NWs)). Actually, in each of these reported cases, a new formation of cell viability loss was recorded even in the dark conditions [13,14]. In our experimental finding, a concentration of the ZnO and Fotolon, and a compatible light source along with a suitable light dose is also a very important factor for cell viability loss control. The basic analogy of cell viability loss under UV irradiation when cells/tissue are exposed with ZnO and Fotolon is perfect for the absorption of UV light by ZnO nanoparticles UV light, and as an emission reaction inside the tissue, which provides a white light broadband light capable of stimulating a significant chemical reaction, which leads to reactive oxygen species (Free radicals and singlet excited oxygen) via the photosensitizer complex with ZnO nanoparticles. In this way, all of the light losses and the significant bioavailability of the drug can be overcome for cell necrosis/killing of cancerous tissues after the achievement of the exact threshold of light and optimal concentration of ZnO and Fotolon in the target/cancerous site [15,16]. It has already been proved by many researchers that nanoparticles and copolymers nanoparticle play an important role for drug delivery therapeutic effectiveness, with minimal side effects [17]. Multiple regression statistical tests were applied on the cellular viability data plotted between darkness, the presence of light (λ ≈ 630 nm), and UV-light. It was observed by statistical analysis that the multiple regression analysis (0.05) depicted a *p* < 0.05 (*p*-value of 0.0173), which is significant. The statistical results validate the accuracy of the experimental data, which of course is in excellent agreement with the experimental plotted data.

**Figure 8:**
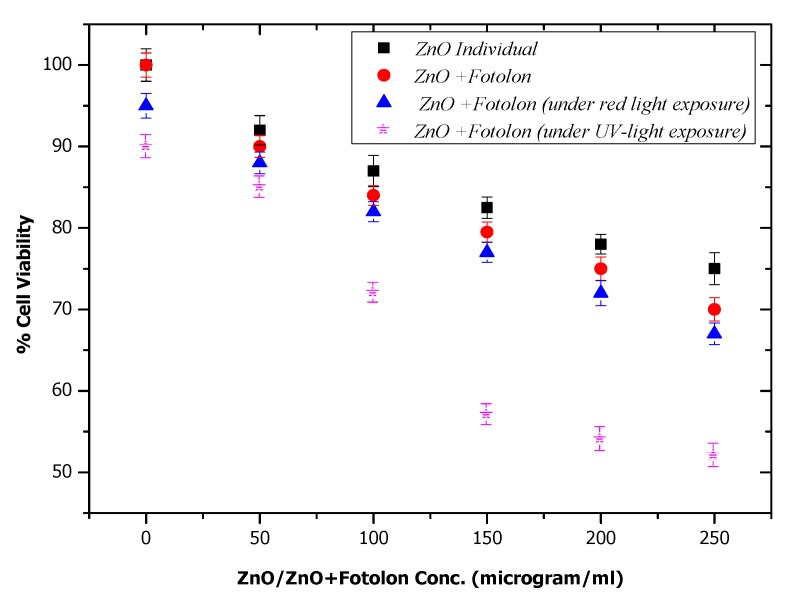
Comparison for % loss in HepG2 cell viability.

In the current study, it was necessary to investigate the actual toxicity mechanism of the individual ZnO nanoparticles and ZnO nanoparticles complexed with Fotolon (Chlorine e6) in darkness and under light exposure. Figure 8 depicts the four different experimental schemes toxicity results. The experiment was performed in an HepG2 cells model in the presence and absence of light (suitable dose). It is clear from the results that the ZnO nanoparticles have insignificant toxic effects in the absence of any drug or light, which is recorded as a 23–25% cell loss, which might be due to a ZnO concentration excess, or to the morphological sharp edge shape of some ZnO nanoparticles. But, when the Fotolon was conjugated with ZnO nanoparticles, and after applying the same experimental parameters in darkness, the cell viability loss approached 30%, which is a satisfactory but not significant value. By extending the frame of the current study, ZnO and Fotolon under exposure to laser red light and UV-light were explored, respectively. A significant difference in the cell treatment purposes of the cell viability loss can be seen in Figure 8, where about 63% and 53% of the cells are viable after systematic photodynamic treatment. A loss in the cell of up to a significant level was recorded in the case of ZnO and Fotolon under UV laser was found. It is clear from the cell viability loss that 20 J/cm^2^ of UV is enough to provide a white light broadband emission spectrum, which consists of red light along with other complementary colors. The red wavelength of light is suitable for stimulating the photochemical reactions and free radicals after interacting with the third-generation photosensitizer Fotolon. Therefore, the cell viability loss is significant in the case of the HepG2 cells labeled with ZnO and Fotolon under a UV laser, compared with those labeled with ZnO and Fotolon under a laser red light. This means that the UV light is being used as an indirect source, which first interacts with ZnO, and then the emitted light can be exposed to Fotolon. There are too many parameters that can be explored and that are under debate, which will be discussed in the future.

### 3.5. Image Analysis and Quantification

Microscopy was performed for HepG2 cells analysis before and after treatment. Histopathological analysis of cell model was quantified by using Image J software. Controlled and treated HepG2 cells were stained with DAPI or 4′,6-diamidino-2-phenylindole and fixed on glass cover slip. The microscope parameters are 40× objectives with a z-step size: 0.5 μm. The images were recorded at a magnification 100×. HepG2 cells selected area in the image was 20.492 μm^2^ with Intensity Mean Value (IMV) 280.2.

### 3.6. Cellular Morphology Analysis

**Figure 9:**
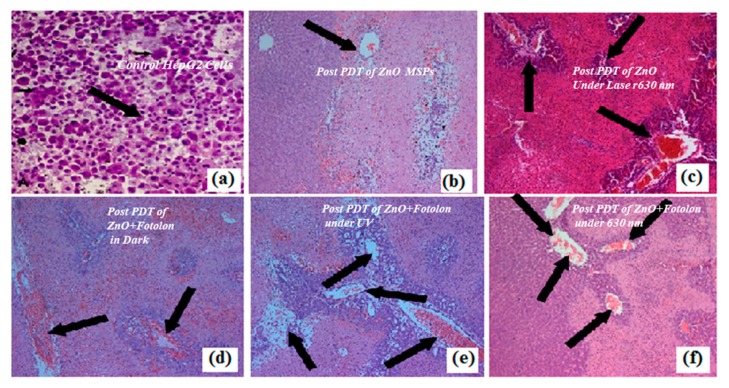
Microscopic snapshots of depth of necrosis before and after PDT Scheme. (**a**) Control liver model with prominent nucleus morphology. (**b**) ZnO nanoparticles toxicity in the dark showing very superficial necrosis. (**c**) Obtained post ZnO nanoparticles treatment and red laser (630 nm) irradiation. (**d**) Obtained post ZnO + Fotolon (chlorine e_6_) treatment in dark. (**e**) Obtained post ZnO + Fotolon (chlorine e_6_) treatment under exposure of UV lamp light. (**f**) Obtained post ZnO + Fotolon (chlorine e_6_) treatment under exposure of red laser (630 nm). The images were recorded at a magnification 100×.

Figure 9 shows microscopic snapshots for the depth of necrosis before and after PDT application. A control liver model with prominent nucleus morphology is demonstrated in Figure 9a, while Figure 9b–f depicts the histopathological data after the PDT procedure for example, when the HepG2cells were exposed with pure ZnO nanoparticles, ZnO nanoparticles complex with Fotolon in dark, and ZnO nanoparticles complex with Fotolon under UV light exposure and red light exposure. The PDT procedure confirmed that the toxicities of both the individual ZnO nanoparticles and their complexes with photosensitizer (chlorine e6) are time and concentration dependent. The Zn/ZnO nanoparticles with a sharp edge (having more probability of cell trauma/stress) and a proper concentration give the high probability of cell necrosis. In the current experiment, it was revealed that the synthesized nanoparticles had the capability of liberating more of the cytotoxicity, which concluded that there was mechanical stress/trauma in cells’ necrosis [18]. From the current experiments and other investigations, we noticed that the optimal time of incubation for the maximum accumulation of a drug into the liver model is between half an hour to four hours, as confirmed from the fluorescence spectroscopy (Nd:YAG, second harmonic laser as light source). In the histopathological analysis, it was clear that the maximum necrosis depths were recorded when suitable concentrations of ZnO and Fotolon were exposed to UV lamp light (20 J/cm^2^), even after 3 h of incubation after microinjection. In Figure 9a, the live nuclei in the control liver model are depicted. In the case of the ZnO nanoparticles’ toxicity in the dark, very superficial necrosis was monitored through the histopathology, as shown in Figure 9b. In the case of the ZnO nanoparticles and red light (630 nm), the results are totally different; it might be possible that red light delivers a threshold value of a dose that is able to produce photochemical reactions in the targeted territory (depicted in Figure 9c). The results in the case of ZnO and Fotolon (chlorine e6) under the exposure of UV-lamp light are very interesting, as the broadband emission of white light pattern takes place from ZnO nanoparticles when directly irradiated with UV light [19]. This white light is a combination of red, blue, and green visible light pattern, and the red light of these emission broadband acts as a photochemical reaction stimulator. Therefore, the maximum tissue debris was counted, as shown in Figure 9d, in hte case of UV illumination tissue labelled with ZnO and Fotolon. In contrast, when the PDT was practiced under the exposure of 630 nm of a red-light wavelength, a superficial level of necrosis was monitored.

### 3.7. Mathematical Modelling Analysis

**Figure 10:**
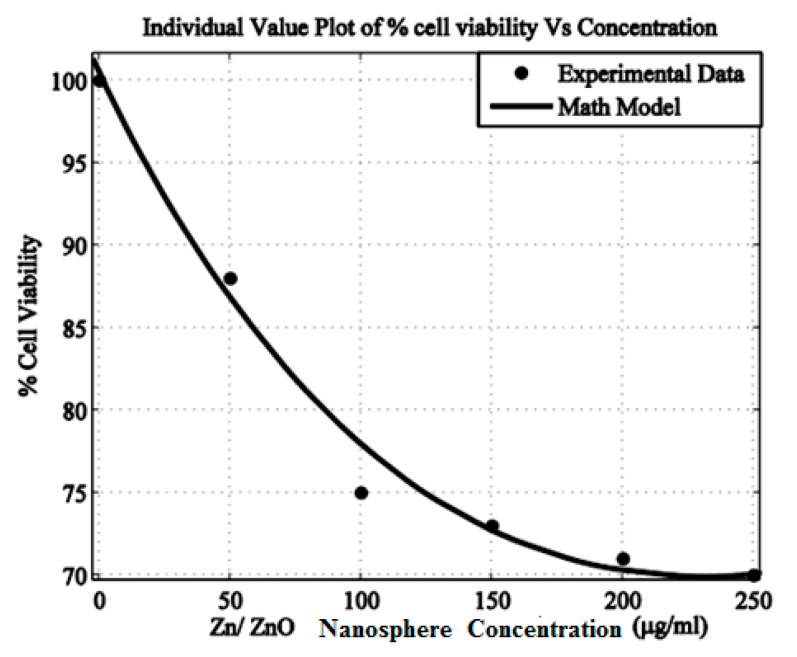
Percent (%) cell viability vs. concentration of Zn nanoparticles.

**Figure 11:**
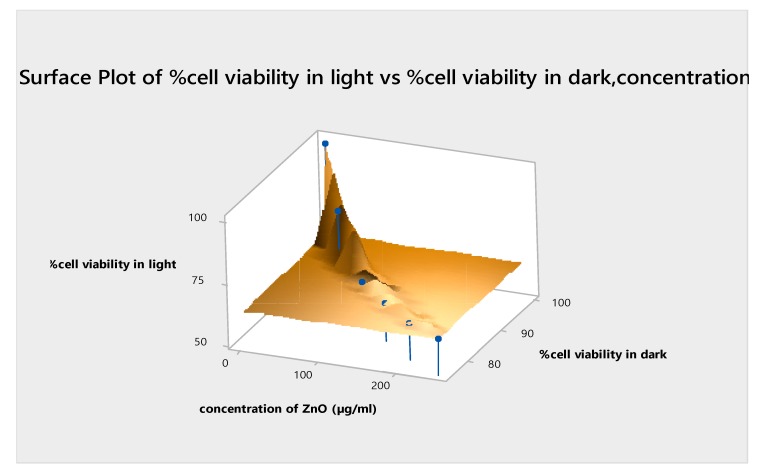
Surface plot for in vitro % cellular viability in the light vs. % cellular viability in the dark (concentration; λ ≈ 630 nm).

The method of least square error is applied to the experimental data in order to have a math model of the dependency. Equation (2) describes the dependency of the cell viability on the concentration of zinc nanoparticles. The values of other parameters are as follows: Sum of Squared Errors (SSE), 9.899; R-square, 0.9862; adjusted R-square, 0.9655; Root Mean Square Error (RMSE), 2.225. The value of R-square (0.9862) indicates the correctness of the math model, which is also evident from the fitness, as shown in Figure 9. A similar trend was observed before, in our published data, when the cytotoxicity of the Ni nanotubes was investigated in the HeLa cell line. Figure 10 shows the coherence agreement of the experimental and modulated data, which depicts the cell viability loss due to the ZnO concentration dependency behavior. In addition, the consistency loss in the cell viability can be seen in Figure 10. A surface plot for cellular loss in light versus the cellular loss in darkness, and the ZnO nanoparticles and Fotolon (chlorine e6) concentration in the HepG2 cell line is given in Figure 11. It is noticeable that the cell loss has increased with the light-based PDT protocol, and it keeps on increasing until it reaches a constant value near a 200–250 µg/mL concentration for zinc oxide nanoparticles with a Fotolon complex. It is concluded that when the cell/tissue model labeled with an optimal concentration of ZnO nanoparticles complex with Fotolon reflects the significant cell viability loss after being exposed with a suitable dose of UV light when compared to the other group’s comparison toxicity, which might be new contribution of ZnO-based research for biomedical researchers. The scientific reason behind this is that ZnO gave a broadband spectrum of emission light, the red part of this emission light plays a role for a photodynamic reaction in the presence of Fotolon (chlorine e6), and the molecular form of oxygen in the vicinity [12]. In the recently reported data, tremendous studies were done regarding the toxicity of ZnO nanoparticles, and comparative toxic effects of TiO_2_ nanoparticles were done, which might be an efficient drug for cancer treatment in the presence of a compatible laser [20,21,22].

(2) Percent (%) cell viability=a×exp(b×x)+c×exp(d×x)

where *x* is the concentration of zinc nanoparticles, while the constants (*a*, *b*, *c*, and *d*) are extracted from the method of least square error, as follows [23,24]:

(3) a=61.7

(4) b=−0.006713

(5) c=38.73

(6) d=0.001676

## 4. Conclusions

In summary, Zn/ZnO nanoparticles were synthesized and characterized, and a successful strategy for applying them in human liver carcinoma was demonstrated. The toxicity/phototoxicity of the recommended nanospheres was tested in the HepG2 cellular model and liver tissue of a Wistar rats model. It was concluded that the toxicity of the synthesized nanoparticles is at a maximum towards the liver tissue model of the Wistar rat after 4 h of iv drug delivery protocol in the presence of 20 J/cm^2^ of UV lamp light irradiation. In the HepG2 cell model, a similar toxicity pattern was explored. 

From the MTT assay and the histopathological data analysis, it was confirmed that the post PDT results of the ZnO nanoparticles exposed to HepG2 under UV laser irradiation is very pleasing, which plays a key role for liver cancer treatment, compared with the pure ZnO nanoparticles labeled to the HepG2 cell, or the Fotolon^®^ and ZnO nanoparticles under a red laser light. We concluded that the HepG2 cells labeled with ZnO and Fotolon under the illumination of UV light depicts the marvelous loss in cell viability, which should be a great symbol towards cancer therapy.

Now, it is time to close the toxicity chapter of ZnO nanomaterials by emphasizing that ZnO is bio-safe and biocompatible, up to certain limit, and can be used as drug delivery vehicles. Above the threshold dose (above 250 µg/mL), its concentration is toxic; some specific cases of toxicity have occurred because of the size and morphological background, and the specifically photodynamic behavior of the ZnO nanoparticles. The current study has highlighted the potential dimensions of applying ZnO nanoparticles in photodynamic therapy. The obtained results are calling for more experiments, which would lead to wider medical applications by, for example, applying photodynamic therapy in conjunction with ionizing radiation therapy, to improve the efficiency of cancer treatment.

## Supplementary Materials

The following are available online at http://www.mdpi.com/2072-666X/10/1/60/s1, Figure S1: PDT Overview of Wistar Rat Model.
