# Correction: Iqbal, S. et al. Empirical Modeling of Zn/ZnO Nanoparticles Decorated/Conjugated with Fotolon (Chlorine e6) Based Photodynamic Therapy towards Liver Cancer Treatment. *Micromachines*, 2019, *10*, 60

**DOI:** 10.3390/mi11030262

**Published:** 2020-03-01

**Authors:** Seemab Iqbal, Muhammad Fakhar-e-Alam, M. Atif, Nasar Ahmed, Aqrab -ul-Ahmad, N. Amin, Raed ahmed Alghamdi, Atif Hanif, W. Aslam Farooq

**Affiliations:** 1 Department of Physics, Government College University, Faisalabad 38000, Pakistan; seemabiqbal11@hotmail.com (S.I.); gourmani5@yahoo.com (N.A.); 2 Key Laboratory of Magnetic Materials and Devices & Division of Functional Materials and Nanodevices, Ningbo Institute of Materials Technology and Engineering, Chinese Academy of Sciences, Ningbo 315201, China; 3 Department of Physics and Astronomy, College of Science, King Saud University, Riyadh 11543, Saudi Arabia; muhatif@ksu.edu.sa (R.a.A.); wafarooq@hotmail.com (W.A.F.); 4 Department of Physics, University of Azad Jammu and Kashmir, Muzaffarabad 13100, Pakistan; fakharphy@outlook.com; 5 School of Physics, Dalian University of Technology, Dalian 116024, China; a.aqrab4469@gmail.com; 6 School of Microelectronics, Dalian University of Technology, Dalian 116024, China; 7 Botany and Microbiology Department, College of Science, King Saud University, Riyadh 11543, Saudi Arabia; ahchaudhry@ksu.edu.sa

In the published paper [1] (https://www.mdpi.com/2072-666X/10/1/60), Figure 9b,e should be corrected as follows:

**Original Figure:**

**Figure 9:**
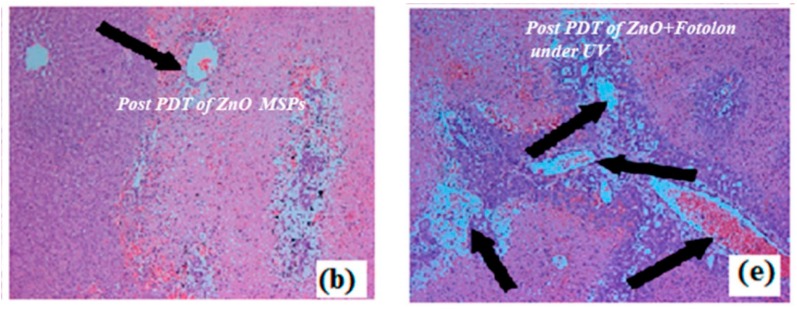
Microscopic snapshots of depth of necrosis before and after PDT Scheme. (**b**) ZnOnanoparticles toxicity in the dark showing very superficial necrosis. (**e**) Obtained post ZnO + Fotolon (chlorine e_6_) treatment under exposure of UV lamp light. The images were recorded at a magnification 100×.

**New Figure:**

**Figure 9:**
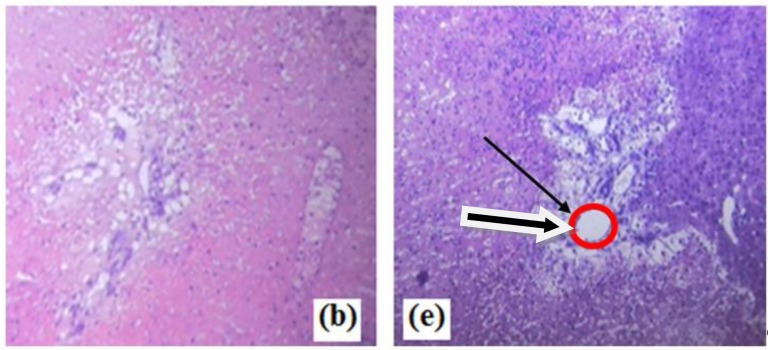
Microscopic snapshots of depth of necrosis before and after PDT Scheme. (**b**) ZnOmicrospheres toxicity in the dark showing very superficial necrosis. (**e**) Obtained significant necrosis when treated with ZnO + Fotolon (Chlorine e_6_) under exposure of UV lamp light. Images were recorded 100× magnification, and black arrow in Figure 9e depicts the region of interest (ROI) and especially necrosis area.

The changes do not affect the scientific results. We apologize for any inconvenience caused to the readers by these errors. The manuscript will be updated, and the original will remain online on the webpage for the article including a reference to this Correction.
