# Supplementary figures and images for: Empirical Modeling of Zn/ZnO Nanoparticles Decorated/Conjugated with Fotolon (Chlorine e6) Based Photodynamic Therapy towards Liver Cancer Treatment
Source: Micromachines (Basel). 2019 Jan 17;10(1):60. doi: 10.3390/mi10010060 (PMC6357181; doi:10.3390/mi10010060)

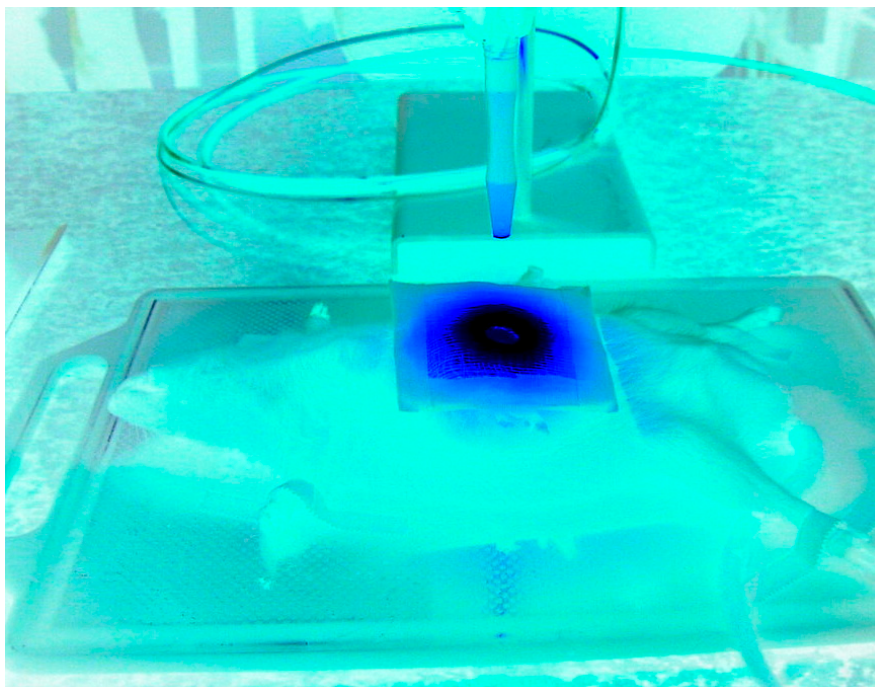

**Figure S1.** Photodynamic therapy (PDT) overview of wistar rat model.

Supplement: Supplementary file 1 [file micromachines-10-00060-s001.pdf]
